# Linear motor driven-rotary motion of a membrane-permeabilized ghost in *Mycoplasma mobile*

**DOI:** 10.1038/s41598-018-29875-9

**Published:** 2018-07-31

**Authors:** Yoshiaki Kinosita, Makoto Miyata, Takayuki Nishizaka

**Affiliations:** 1 Department of Physics, Gakushuin University, 1-5-1 Mejiro, Toshima-ku, Tokyo, 171-8588 Japan; 2 Graduate School of Science, Osaka City University, 3-3-138 Sugimoto, Sumiyoshi-ku, 8, Osaka, 558-8585 Japan; 3 Present Address: Institute of Biology II, Freiburg University, Schaenzlestreet 1, 79104 Freiburg Germany; 4 The OCU Advanced Research Institute for Natural Science and Technology, Osaka City University, Osaka, Japan

## Abstract

*Mycoplasma mobile* exhibits a smooth gliding movement as does its membrane-permeabilized ghost model. Ghost experiments revealed that the energy source for *M. mobile* motility is adenosine triphosphate (ATP) and that the gliding comprises repetitions of 70 nm steps. Here we show a new motility mode, in which the ghost model prepared with 0.013% Triton X-100 exhibits directed rotational motions with an average speed of approximately 2.1 Hz when ATP concentration is greater than 3.0 × 10^−1^ mM. We found that rotary ghosts treated with sialyllactose, the binding target for leg proteins, were stopped. Although the origin of the rotation has not been conclusively determined, this result suggested that biomolecules embedded on the cell membrane nonspecifically attach to the glass and work as a fluid pivot point and that the linear motion of the leg is a driving force for the rotary motion. This simple geometry exemplifies the new motility mode, by which the movement of a linear motor is efficiently converted to a constant rotation of the object on a micrometer scale.

## Introduction

**Figure 1:**
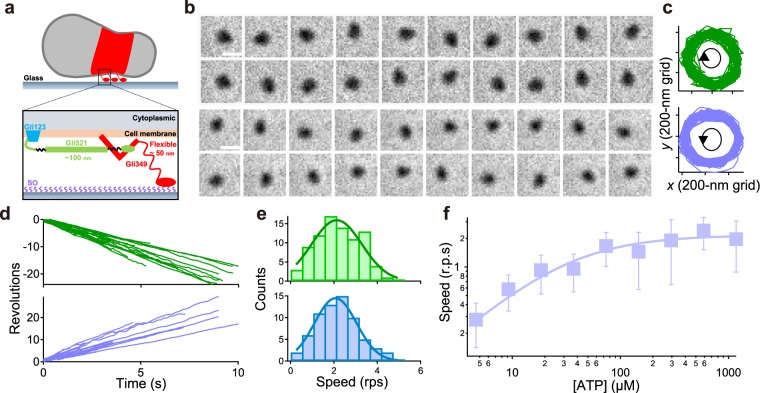
ATP-dependent rotation of tethered ghosts. (**a**) Schematics of a single cell (*top*) and a gliding machinery (*bottom*). (**b**) *Top* and *bottom*: At 100 ms intervals, sequential images of a ghost in CW and CCW rotation, respectively. Scale bar,1 μm. (**c**) The trajectories of the center of mass in CW and CCW rotation. Blue and green represent CW and CCW rotation, respectively, and their colors coincide with d and e. (**d**) Time course of the revolution of the tethered ghost. (**e**) Histograms of speed in both directions at 295, 590, and 1180 μM [ATP]. Solid lines represent the Gaussian function, where 2.1 ± 1.2 rps and 2.1 ± 0.9 rps in CW and CCW rotations, respectively (n = 84 in CW rotation, n = 66 in CCW rotation). (**f**) The rotation rate of ghosts at different [ATP]s (n = 480). Solid line showed the Michaelis-Menten kinetics: *V* = *V*_max_ [ATP]/(*K*_m_ + [ATP]), where *V*_max_ and *K*_m_ were 2.2 Hz and 32 μM, respectively.  Data are representative of at least three independent experiments.

*Mycoplasma mobile* (*M. mobile*) is a flask-shaped bacterium that can smoothly glide on a solid surface in the direction of a protrusion at a speed of up to 4.5 μm s^−1^ ^1^ (Fig. 1a *top*). Genomic sequencing and analysis have revealed that the mechanism must differ from those of other forms of motor protein systems and bacterial motility, as *M. mobile* lacks bacterial flagella and genes encoding conventional motor proteins such as myosin and kinesin^2^. Proteins essential for the gliding movement comprise surface and internal structures. The surface structure is composed of three proteins, and named as Gli123, Gli349, and Gli521 (Fig. 1a *bottom*). These proteins essential for gliding are localized at a cell pole, and each of their numbers is estimated to be approximately 450^3,4^. Gli349 extends out from the cell membrane and has a rod-like structure, approximately  95 nm in total, with two flexible hinges when isolated. The series of studies with mutants and inhibitory antibodies suggested that Gli349 works as a “leg” by binding to and releasing from a substrate covered with randomly arranged sialylated oligosaccharides (SOs)^5,6^. The monoclonal antibody against Gli521 caused cells to stop on the solid surface; therefore, Gli521 with a size of 120 nm is assumed to function as a crank^7,8^. The Gli123 mutant affected the distribution of Gli349 and Gli521 proteins, suggesting that Gli123 might be essential as a scaffold for other molecular machineries^9^. The internal structures including the α-and β-subunit homologs of the F-type ATPase co-localized on the gliding machineries, suggesting that the internal structure might function as the motor for *Mycoplasma* gliding^10,11^. Notably, the membrane-permeabilized ghost model revealed that the gliding machineries are driven by ATP hydrolysis^12^. With this information, the “centipede” model has been proposed, wherein Gli349 repeatedly catches, pulls, drags, and releases SOs driven by ATP hydrolysis^3,4^. In theory, hundreds of surface structures should cooperate and work simultaneously for smooth movement^13^. On the other hand, an elongated cell exhibited the pivoting movement, suggesting that gliding units are working independently, rather than cooperatively, to propel the cell forward^14^. Recently, the discrete 70 nm steps have been detected under the designed condition that the working leg number was controlled by the addition of free sialyllactose (SL), which might correspond to the strokes of a single gliding unit^15^. Additionally, the force with a size of 1–2 pN was measured using optical tweezer, which is possibly attributable to the single-gliding unit force^16^. However, there remains the possibility that the stepping behavior comprises several leg proteins. To explore the mechanical insights of a single gliding unit, an advanced assay is required. Here, we report the novel ‘tethered-ghost’ assay to extract the rotary motion of *M. mobile* instead of a gliding motion. By treating cells with 0.013% Triton X-100, ghosts exhibited a rotation at a fixed position like a tethered cell in flagellated bacteria^17–19^. Although we could not determine the number of gliding units involved in this rotation, we characterized the motor torque and energy-conversion efficiency using the tethered-ghost assay.

## Results

### ATP-dependent rotation of rotary ghosts

To explore the motor function in greater detail, we constructed a motility assay that enabled the detection of rotary ghosts. Previously, the membrane-permeabilized exhibited model was prepared with 0.009% Triton X-100, and most ghosts showed a gliding motion after the addition of ATP^12,15^. In contrast, we found that a few percentage of ghosts prepared with 0.013% Triton X-100 rotated at a fixed position like tethered-flagellated bacteria^17–19^ (Supplementary Movie 1). Under this condition, we also detected the gliding motion in approximately 50% of the ghost population, and the gliding speed was similar to that of live cells at saturated [ATP]s (Fig. S2).

Rotational motions occurred in both directions, and the population of each cell was 56% in the CW and 44% in the CCW direction (n = 150; Fig. 1b). Note that the center position of the rotation did not move and that the radius also remained constant, indicating that some flexible part was attached to the glass surface (Fig. 1c). Rotational rate was calculated from the slope of the revolution (Fig. 1d). The rotational rate was determined as 2.1 ± 1.2 rps in the CW direction, and 2.1 ± 0.9 rps in the CCW direction at 295, 590, and 1180 μM ATP (n = 84 in CW, n = 66 in CCW; Fig. 1e). We did not observe any differences between the CW and CCW rotational rates (*P* = 0.9418 > 0.05 by *t*-test). Therefore, we did not distinguish CW and CCW rotations and analyzed them collectively.

We then investigated the effect of ATP concentration on the rotational rate of ghosts. In the range of 4–1200 μM [ATP]s, the relationship between a rotational rate and [ATP] obeyed simple Michaelis-Menten kinetics, where *V*_max_ and *K*_m_ were 2.2 Hz and 32 μM, respectively (n = 480; Fig. 1f). This suggested that the motor had no cooperativity in the ATP binding event. Note that the discrete steps were detected under low [ATP]s, suggesting that ATP binding is the rate-limiting step for rotary movement in this condition (Fig. S3); in addition, the high-speed imaging enabled us to detect stepwise motions even at 3.5 × 10^−1^ mM ATP, as described in a later section.

**Figure 2:**
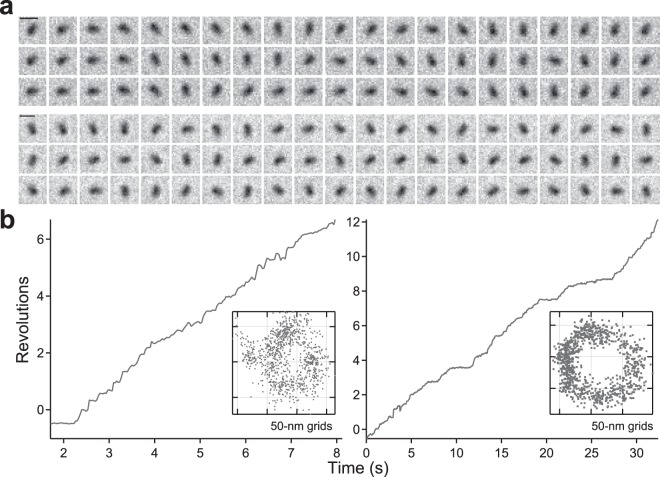
Propeller rotation. (**a**) Sequential phase-contrast images of a propeller rotation at 100 ms intervals under 8 μM (*top*) and 32 μM [ATP] (*bottom*). The rotation axis was located at the middle of the ghost and rotated like a propeller. Scale bar, 1 μm. (**b**) The time course of revolutions under 8 μM (*left*) and 32 μM [ATP] (*right*). *Inset*: *x*-*y* trace. Data are representative of at least three independent experiments.

Most of the rotating ghosts had their rotational axis at one end of body, i.e., the tip or behind of body as shown in Fig. 1b. Meanwhile, the rotational axes of some ghosts were located in the middle and rotated like a propeller (Supplementary Movie 2 and Fig. 2), suggesting that the real rotary motor binds to the glass surface (see Discussion).

### Reduction of rotational rate by addition of binding target for leg proteins

**Figure 3:**
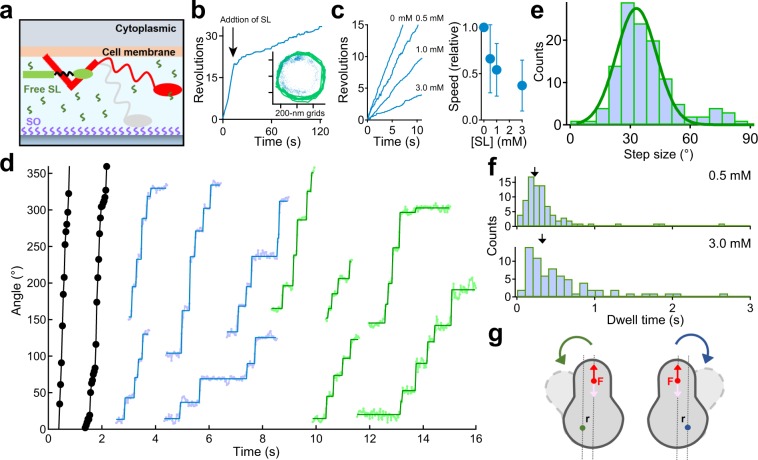
Linear movement generated the rotary motion. (**a**) Schematic illustration of the experiment. Gray and red legs are before and after the addition of sialyllactose (SL), respectively. (**b**) Typical example of the time course of a revolution in the presence of a free SL in solution. The arrow represents the time when SL was added. *Inset*: Green line and blue dot at 33 ms intervals represent a rotational trace before and after the addition of free SL in solution, respectively. (**c**) Dependence of rotation rate on the concentration of free SL at the 1 mM [ATP]. *Left*: Time course of revolution under the various [SL]s, 0.5–3.0 mM. *Right*: The relationship between the rotational speed and mM [SL] (n = 56). The relative speed *f* represents *f*_after_/*f*_before_. (**d**) Typical examples of a stepwise rotation under the various [SL]s. Black, cyan, and green dots represent the raw data at 0 mM [SL], 0.5 mM [SL], 3.0 mM [SL], respectively. Rectangles in the rotation are lines fitted by the step-finding algorithm (see Methods). (**e**) Histogram of the step size calculated by a step-finding algorithm (n = 125). Solid line showed the Gaussian function, where the size was 33.1 ± 10.1°. (**f**) Histograms of dwell times at 0.5 mM [SL] and 3.0 mM [SL]. Arrow in each graph indicates the average value which are 0.37 ± 0.39 s in 0.5 mM and 0.55 ± 0.51 s in 3.0 mM, respectively (n = 83 in 0.5 mM, n = 71 in 3.0 mM). (**g**) Schematics of the pivoting model for rotation. The green and blue dots represent the tethered point to the surface. The red dot represents the position of active leg(s); additionally, the red and pink arrows correspond to the direction of thrust from a surface and the direction of the leg’s force to a surface, respectively. The width of the gray-dot lines (r) represents the distance between the power stroke of the active leg and tethered point. Depending on the tethered point, the rotational direction could be changed; e.g., the CW direction in the case of the blue tethering point.  Data are representative of at least three independent experiments.

To investigate whether the leg protein contributed to the rotation, we treated ghosts with SL, a binding target for legs^20,21^ (Fig. 3a). Notably, rotary ghosts did not detach from the glass surface but slowed down and/or stopped, while gliding ghosts detached from the surface (Supplementary Movie 3, Fig. 3b). Additionally, the ratio of rotational rate (*f*_after_/*f*_before_) decreased with [SL] (Fig. 3c). In this condition, the discrete stepwise movements were also detected even at 1 mM ATP, which might correspond to the binding time of the leg to fixed SOs on a glass surface (Fig. 3d). To determine the stepping angle quantitatively, we next fitted the data with a step-finding algorithm (see Methods). From this analysis, the step size was estimated to be 33.1 ± 10.1°, assuming that the histogram comprises a single peak (Fig. 3e; n = 125). The dwell time between steps depended on [SL], where the average and standard deviation (SD) were 0.37 ± 0.39 in 0.5 mM and 0.55 ± 0.51 s in 3 mM, respectively (n = 83 in 0.5 mM, n = 71 in 3 mM; Fig. 3f). This result suggested that the leg binds to free SL in solution and consequently, could not produce the thrust for rotation. From results in this experiment, we proposed the pivoting model for rotation: the leg produces the thrust for rotation, while a flexible point such as the membrane is tethered to the surface (Fig. 3g, see Discussion).

### Effect of antibodies of a gliding machinery on rotation

We next explored the effect of the antibody on rotation. We used monoclonal antibodies (MAb) MAb7 and MAbR19 against Gli349 and Gli521, respectively, which influence the binding activity and gliding speed, respectively^22^. The rotary ghosts that were treated with MAb7 stopped the rotation but did not detach from the glass. On the other hand, the gliding ghosts treated with MAb7 dissociated from the glass surface. In the MAbR19 experiments, both gliding and rotary ghosts stopped suddenly, but did not detach from the glass (Supplementary Movie 4). As stated previously, these results indicated that the crank Gli521 transmits the force, and the leg(s) Gli349 binds to and releases from SOs and produces the thrust^3,4^.

### Detection of stepwise rotation under various [ATP]s

**Figure 4:**
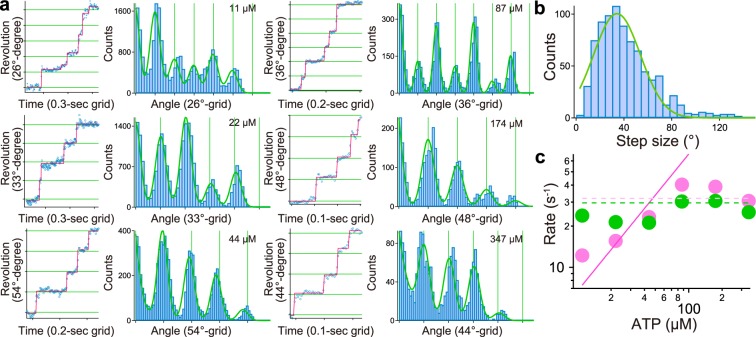
Stepwise rotation. (**a**) *Left*: The red line represents the fitting of the raw data by a step-finding algorithm. *Right*: Histograms of the pairwise distance function of the raw data. Green solid lines represent the sum of Gaussians. (**b**) Histograms of the step size extracted by a step-finding algorithm in the range of 11–347 μM ATP. Solid lines represent the single Gaussian, where the peak and SD were 34.2 ± 19.5° (n = 841). (**c**) The relationship between rate constants and [ATP]. Green and pink dots represent the rate constants estimated by the exponential fit to individual data according to the follow equation: constant × (exp(−*k*_1_ × t) − exp(−*k*_2_ × t)), where *k*_1_ and *k*_2_ represent green and pink filled circle in the graph. (see also Fig. S6). Pink and green dot lines represent the rate constants obtained by the global fitting using the following equation: constant·(exp(−(*k*_on_ × [ATP] × t) − (exp(−*k*_1_ × t) for 11–44 μM ATP and constant·(exp(−*k*_1_ × t) − exp(−*k*_2_ × t)) for 87–347 μM ATP, where *k*_on_ was the rate constant for ATP binding (6.7 × 10^5^ M^−1^ s^−1^) and two ATP-independent reactions with *k*_1_ and *k*_2_ as 29.6 s^−1^ and 31.9 s^−1^. The solid pink line represents an ATP binding constant acquired by the analysis of global fit. Data are representative of at least three independent experiments.

We successfully detected the stepwise rotation under low [ATP]s and in the presence of SL. To gain mechanical insight into the motility mechanism under various [ATP]s, we next performed high-speed imaging of rotations with a time resolution of 4 ms. Remarkably, we also detected the intermittent pauses at 347 μM ATP (Fig. 4a). The 178 time courses in 40 ghosts showed the instantaneous stepping motion and could be successfully fitted by a step-finding algorithm (outputs are superimposed as a line in Fig. 4a). We could not detect any differences in step angles in the range of 11–347 μM [ATP] (Fig. S4). The histogram of step angles under various [ATP]s comprises a single peak, where the peak and SD were 34.1 ± 19.6° (Fig. 4b; n = 841).

With the angular velocity and viscous friction of a single ghost, the stepping torque *T* under various [ATP]s could be estimated as the following equation: *T* = the angular velocity × the viscous friction of ghost (2.4 pN nm s). To check whether the stepping torque was dependent on [ATP], we then investigated the angular velocity between steps. We extracted consecutive steps of 30–40° from the rotational record at each [ATP], and each step was averaged and superimposed as the thick cyan line in Fig. S5. To eliminate the contribution of short pauses, we calculated the slope between 4–30° with a linear fit. From this analysis, the angular velocity was estimated to be 35–50 rad s^−1^ under various [ATP]s (Fig. S5). Since the ghost’s shape did not change under various [ATP]s, this result indicated that ghosts produced constant torque irrespective of [ATP]. From this analysis, the stepping torque was estimated to be 85–120 pN nm, which was a few times larger than F_1_-ATPase^23^, but 10 times smaller than the bacterial flagellar motor^24^.

### Characterization of dwell time

To realize the molecular mechanism in terms of chemo-mechanical coupling, we next extracted the dwell time with the step-finding algorithm. At [ATP] < *K*_m_ = 32 μM, the distribution of dwell time showed double exponential decay, indicating that the stepwise motion comprised two chemical reactions. To determine the rate constant of individual reactions, histograms were fitted with a double exponential: const·(exp(−*k*_1_ × t) − exp(−*k*_2_ × t)) (see also Fig. S6). Within the range of 11–44 μM ATP, *k*_1_ increased linearly with external [ATP]s, indicating that *k*_1_ was the rate of ATP binding (pink circles in Fig. 4c). Even at [ATP] > *K*_m_, histograms showed a double exponential decay, suggesting that two reactions of ATP-independent manners could exist, such as the Pi release and binding time of Gli349 to the SOs. Histograms were fitted with a double exponential at [ATP] > *K*_m_, where two rate constants were around 30 s^−1^, which was similar to the value of the ATP-independent rate at low [ATP] (green circles in Fig. 4c). To describe the chemical reaction at all [ATP]s, a global fit was applied to all histograms: const·(exp(−*k*_on_ × [ATP] × t) − exp(−*k*_1_ × t)) at 11–44 μM ATP and const·(exp(−*k*_1_ × t) − exp(−*k*_2_ × t)) at 87–347 μM ATP, where *k*_on_ for ATP-dependent reactions, and *k*_1_ and *k*_2_ for ATP-independent reactions were calculated to be 6.7 × 10^5^ M^−1^·s^−1^, 29.6 s^−1^ and 31.9 s^−1^, respectively. The rate constant of ATP-independent reactions was similar to that from a previous report, but the ATP-binding rate was 20 times smaller^15^. This discrepancy might be due to the reduction in active legs for the movement treated with a high concentration of detergent. This interpretation coincides with reports on other molecular motors where dwell time was proportional to the number of active motors^25^.

## Discussion

In this study, we established an assay to extract the rotary motion in a gliding bacterium, *Mycoplasma mobile*. With this technique, the ATP-dependent rotation could be observed, and the *V*_max_ and *K*_m_ were estimated to be 2.2 Hz and 32 μM, respectively (Fig. 1f). By applying high-speed video microscopy for observation, we also detected a 34°-stepwise movement and calculated the angular velocity as 35–50 rad s^−1^, which corresponded to 6–8 Hz. In the dwell-time analysis, the two ATP-independent rate constants were estimated to be approximately 30 and 32 s^−1^, indicating that the rate-limiting steps would be 15.5 s^−1^. The average rotational rate was thus calculated with the following equation: *V* = Step size × rate = 34° × 15.5 s^−1^ = 527° s^−1^, corresponding to 1.5 Hz, which roughly coincided with the maximum speed calculated by Michaelis-Menten kinetics (Fig. 1f). We thus concluded that the above discrepancy was due to the inclusion of the dwell time in the analysis in the video-rate observation.

Although the number of gliding units involved in the rotation was not conclusively determined, we inferred the possible motility mechanism of *M. mobile*. From the result that ATP-dependent rate roughly showed the linear-dependent manner, each step might comprise the single turnover of ATP (Fig. 4c). Assuming that one ATP molecule is consumed per step, the mechanical work can be estimated to be 50–70 pN nm in the following equation: *W* = step size (34°) × stepping torque (85–120 pN nm). With this 1:1 scenario, the energy-conversion efficiency was roughly 50–70%, which supplies chemical energy of 100 pN nm when hydrolyzed.

We considered three possible mechanisms to explain the rotation. One scenario is the pivoting model: the leg produces the thrust for the rotation, while a flexible point such as the membrane is tethered to the surface (Fig. 3g). For a distance *r* between the power stroke of the active leg and the tethered point to the glass, the torque was calculated by the following equation: *T* = *r* × *F*, where *T* is the torque, and *F* is the thrust of the leg^26^. With this model, bidirectional rotation could be produced depending on the geometry between the tethering point and the active leg. Given this model and assuming that a step length is 70 nm^15^, the distance *r* was estimated to be 120 nm using the following equation: *L* = 2 *r* sin (*θ*/2), where *L* is a step length, and *θ* is a step angle (34°), which might correspond to the periodicity of gliding machineries. Although the number of legs involved in rotation was not conclusively determined, the thrust could be estimated to be 0.7–1 pN from the above equation, assuming that the number of legs for rotation was driven by a single leg, which was comparable to the values calculated by optical tweezers^16^.

The second rotary model assumes that one part of the same gliding machinery, such as Gli521, directly binds to a glass surface while pulling another part of the complex such as Gli349. If the gliding machinery exhibits constant displacement and force, the various outputs will be detected depending on the geometry of the pivoting model, e.g., the detection of smaller output when *r* is small and vice versa. Considering that the repetitive steps are clear, and the distribution of steps was narrow (Figs 4a and S4), this model might also be possible.

Although the SL experiment might support above two rotary models (Fig. 3), we could not exclude the third rotary model that the internal structure of *M. mobile* directly binds to a glass surface and produces the rotary motion, which is the homolog of F-Type ATPase and co-localized on the gliding machinery^10,11^. This is because some ghosts had a rotation axis in the middle of a single ghost and rotated like a propeller, the centroid of which is at the middle of the ghost, suggesting that the real rotary motor was connected to the glass surface as shown in F_1_-ATPase and bacterial flagella^17–19,22,27^ (Supplementary Movie 2 and Fig. 2a). To address the above possibility that the homolog of F-type ATPase binds to surface and produces a rotation, the stepwise rotation depending on the structural symmetry should be detected in the propeller rotation, i.e., the 120°-steps as seen in F_1_-ATPase^23^. So far, we have detected step-like motions—though not 120°— at a few μM range of [ATP] (Fig. 2b). However, we inferred that the next chemical cycle would start before the completion of mechanical work in this condition. To address whether third scenario is true, therefore, we should perform the experiment in the range of nM [ATP] like F_1_-ATPase for detecting steps^23^. If F-type ATPase is the motor for *Mycoplasma* gliding, the molecular motor through which rotary motion is converted to linear motion might be a common characteristic among gliding bacteria, as demonstrated in *Flavobacterium johnsoniae*^28^.

## Material and Methods

### Strains and cultivation

*M. mobile* strain (ATCC 43663) was grown in Aluotto medium [2.1% (wt/vol) heart infusion broth, 0.56% yeast extract, 10% (vol/vol) horse serum, 0.025% thallium acetate, and 0.005% ampicillin] at 25 °C until the absorbance at 600 nm reached 0.06–0.10^29^.

### Tethered ghost assay

The preparation of ghosts was as previously described, with the exception that all buffers contained 0.1% methylcellulose (M0512; Sigma Aldrich) to prevent ghosts from dissociating from the glass surface^12,15^. The final buffer was composed of 10 mM Tris-HCl at pH 7.5, 50 mM NaCl, 1 mM DTT, 1 mM EGTA, 2 mM MgCl_2_, 0.5 mg ml^−1^ bovine serum albumin, and Mg^2+^-ATP with an ATP-regenerating system (0.2 mg ml^−1^ creatine kinase and 0.8 mg ml^−1^ creatine phosphate) but without methylcellulose. To avoid the depletion of ATP, each experiment was done within 30 min. All experiments were done at RT.

To check the effect of the free sialyllactose (A0828; Sigma Aldrich) and antibodies on the rotation, the 20-ml volume of final buffer containing these reagents was induced into the chamber. The final concentration of antibodies was ~5 μg ml^−1^.

### Microscope

Tethered ghosts were observed under a phase-contrast microscope (IX71; Olympus) equipped with a 100× objective (UPLSAPO 100 with Ph and 1.4 N.A.; Olympus), a CCD camera (VCC-H1600 or LRH1540N; Digimo), a highly-stable customized stage (Chukousha), and an optical table (RS-2000; Newport)^30^. Ghost position was determined by a centroid fitting or ellipsoid fitting with 8-nm accuracy^15,30,31^ (Fig. S1).

### Data analysis

Sequential images were captured as 8-bit images with CCD camera at 4- or 33-ms temporal resolution and converted into a sequential TIF file without any compression.

Torque caused by rotational frictional drag was estimated using the following equation: *T* = *ω*ξ, where *T* is the rotational torque, *ω* is an angular velocity, and *ξ* is a viscous friction. The viscous friction is, for the case of a spherical ghost, given by 8πηa^3^ + 6πηar^2^, where *η* is the viscosity, *a* is the radius of the ghost, and *r* is the radius of rotation. We frequently observed an ellipsoidal ghost, like two spherical ghosts combined. In this case, the viscous friction is given by 2 × 8πηa^3^ + 6πηar_1_^2^ + 6πηar_2_^2^, where r_1_ and r_2_ are the radii of the revolution of the inner and outer ghosts, respectively^32^. The radius of a ghost was measured using ImageJ 1.48 v software (http://rsb.info.nih.gov/ij/). The average viscous friction was estimated to be 2.4 pN nm s.

To determine the stepping angle quantitatively, we used two processes as follows. To roughly extract more than four steps with a regular size of steps, pairwise distance function (PDF) analysis was first applied^15^. We analyzed 129 ghosts at the various [ATP]s, and 267 runs in 44 ghosts were shown to have more than four steps. Typical examples of PDF analysis were shown in Fig. 4a. Next, to extract the instantaneous stepping motion, we applied the step-finding algorithm to 44 ghosts^15,33^. Because the instantaneous stepping motion was only fitted with a step-finding algorithm, we inferred that the multiple legs-driven motion could be excluded in this analysis. A step-finding algorithm was applied to 267 runs in 44 ghosts, and 178 time courses in 40 ghosts were successfully extracted (Fig. 4a).

To estimate the angular velocity *ω*, we selected the 30°–40° steps and averaged the consecutive steps for 0.2 s^27^. Time zero for each step recorded was assigned closest to 15°. To eliminate the contribution of short pauses, we applied the linear fitting to the averaged step between 4° and 30°, where the slope corresponded to the angular velocity (Fig. S5). Using this value, the stepping torque was given by the previously mentioned equation.

## Electronic supplementary material

Supplementary Information

Supplementary Video 1

Supplementary Video 2

Supplementary Video 3

Supplementary Video 4
