# Publisher Correction: Linear motor driven-rotary motion of a membrane-permeabilized ghost in *Mycoplasma mobile*

**DOI:** 10.1038/s41598-018-31113-1

**Published:** 2018-08-21

**Authors:** Yoshiaki Kinosita, Makoto Miyata, Takayuki Nishizaka

**Affiliations:** 1 Department of Physics, Gakushuin University, 1-5-1 Mejiro, Toshima-ku, Tokyo, 171-8588 Japan; 2 Graduate School of Science, Osaka City University, 3-3-138 Sugimoto, Sumiyoshi-ku, 8, Osaka, 558-8585 Japan; 3 Present Address: Institute of Biology II, Freiburg University, Schaenzlestreet 1, 79104 Freiburg, Germany; 4 The OCU Advanced Research Institute for Natural Science and Technology, Osaka City University, Osaka, Japan

Correction to: *Scientific Reports* 10.1038/s41598-018-29875-9, published online 31 July 2018

The original HTML version of this Article contained errors in the naming of the four Supplementary Video files. This error has now been corrected in the HTML; the PDF version of the paper was correct from the time of publication.

