# Supplementary material for: Linear motor driven-rotary motion of a membrane-permeabilized ghost in Mycoplasma mobile
Source: Sci Rep. 2018 Jul 31;8:11513. doi: 10.1038/s41598-018-29875-9 (PMC6068192; doi:10.1038/s41598-018-29875-9)
Supplement: Supplementary file 1 — Supplementary Information [file 41598_2018_29875_MOESM1_ESM.pdf]

Linear motor driven-rotary motion of a membrane-permeabilized ghost in *Mycoplasma mobile*

Yoshiaki Kinosita[1,*], Makoto Miyata[2,3] & Takayuki Nishizaka[1]

[1]Department of Physics, Gakushuin University, 1-5-1 Mejiro, Toshima-ku, Tokyo 171-8588, Japan.

[2]Graduate School of Science, Osaka City University, Sumiyoshi-ku, Osaka 558-8585, Japan

[3]The OCU Advanced Research Institute for Natural Science and Technology, Osaka City University

* Present address: Institute of Biology II, Schaenzlestreet 1, 79104 Germany

This file includes:

Supplementary Figures 1-6

Captions for Supplementary Movies

Supplementary figures

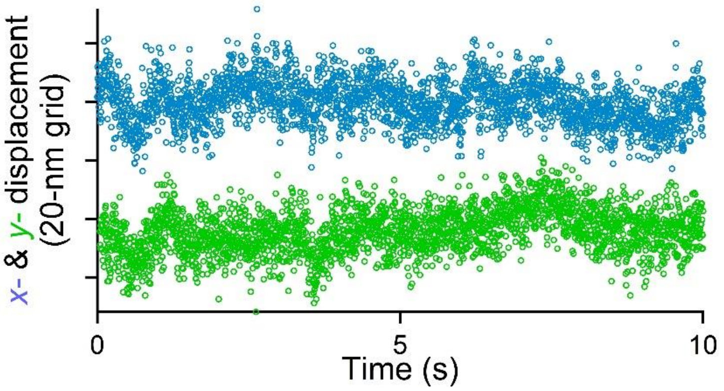

Supplementary Figure 1 Stability and spatial resolution of our setup

Immobilized ghosts on a glass surface were captured with a temporal resolution of 4 ms under a phase-contrast microscope. The position of each cell was determined by a centroid fitting. The SDs of x and y were 7.7 and 7.5 nm, respectively, which corresponded to a spatial resolution of our setup (n = 4).

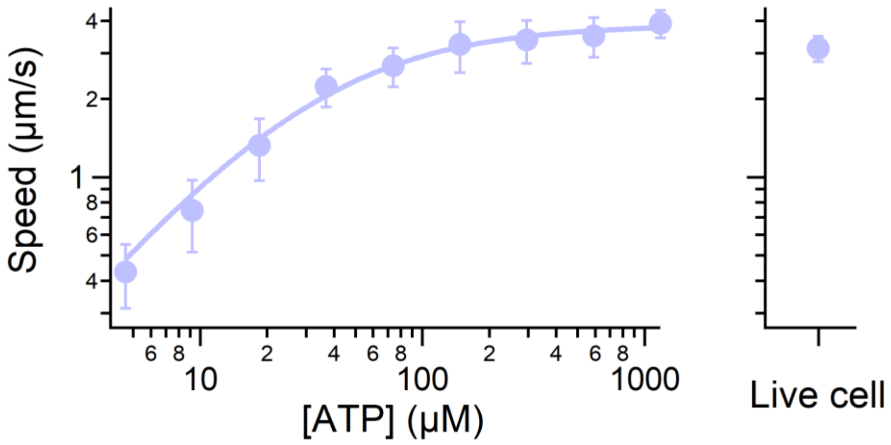

Supplementary Figure 2 Gliding speed of ghosts at different [ATP]s

Left: Data points represent an average gliding speeds of ghosts under various [ATP]s (n = 172). Solid line shows V=V_{max}[ATP]/([ATP]+K_m), where V_{max} and K_m are 3.9 µm s^{-1} and 32 µM, respectively. *Right*: The speed of live cells (3.1 ± 0.4 µm s^{-1}, n = 20).

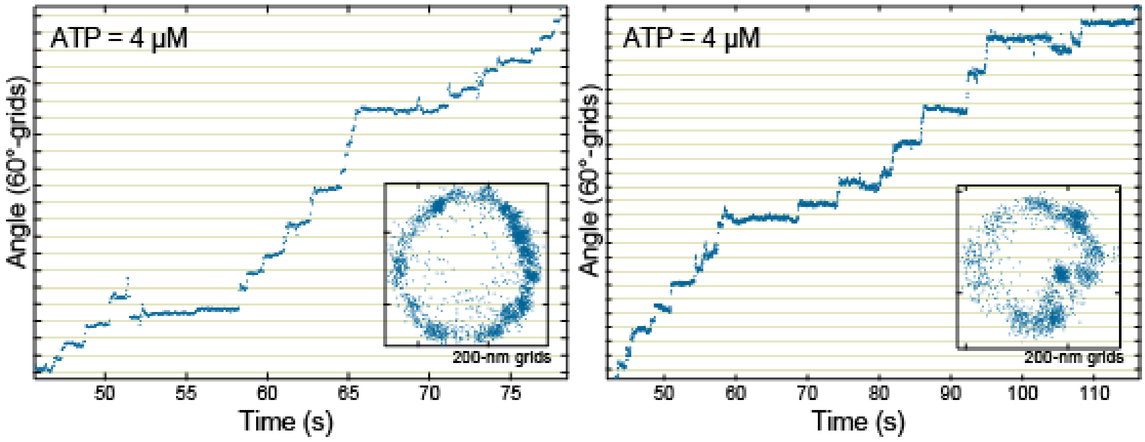

Supplementary Figure 3 Stepwise rotation at low [ATP]

Inset: *x*-*y* trace.

.

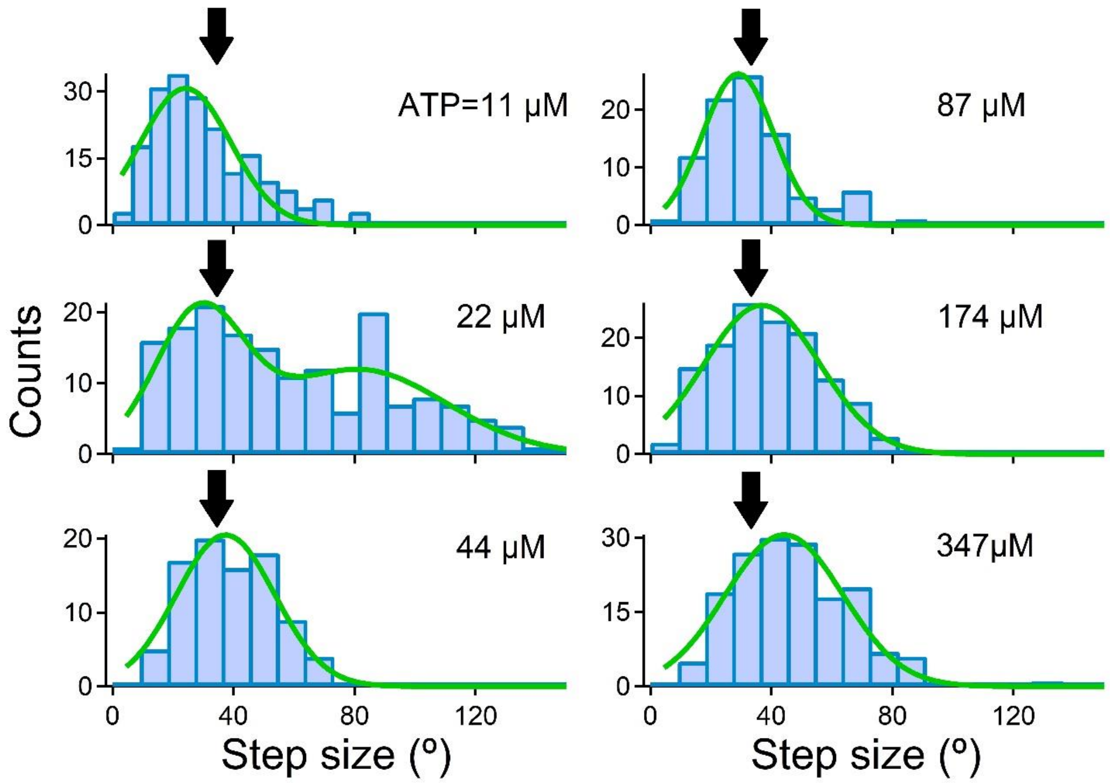

Supplementary Figure 4 Histograms of step angle extracted by a step-finding algorithm under various [ATP]s

Solid lines represent the single or the sum of Gaussian(s). Allow indicates the average value of all data with a size of 34° (n = 841). The number of steps is 198, 169, 89, 92, 131, and 162 steps at 11 μM, 22 μM, 44 μM, 87 μM, 174 μM, and 347 μM, respectively.

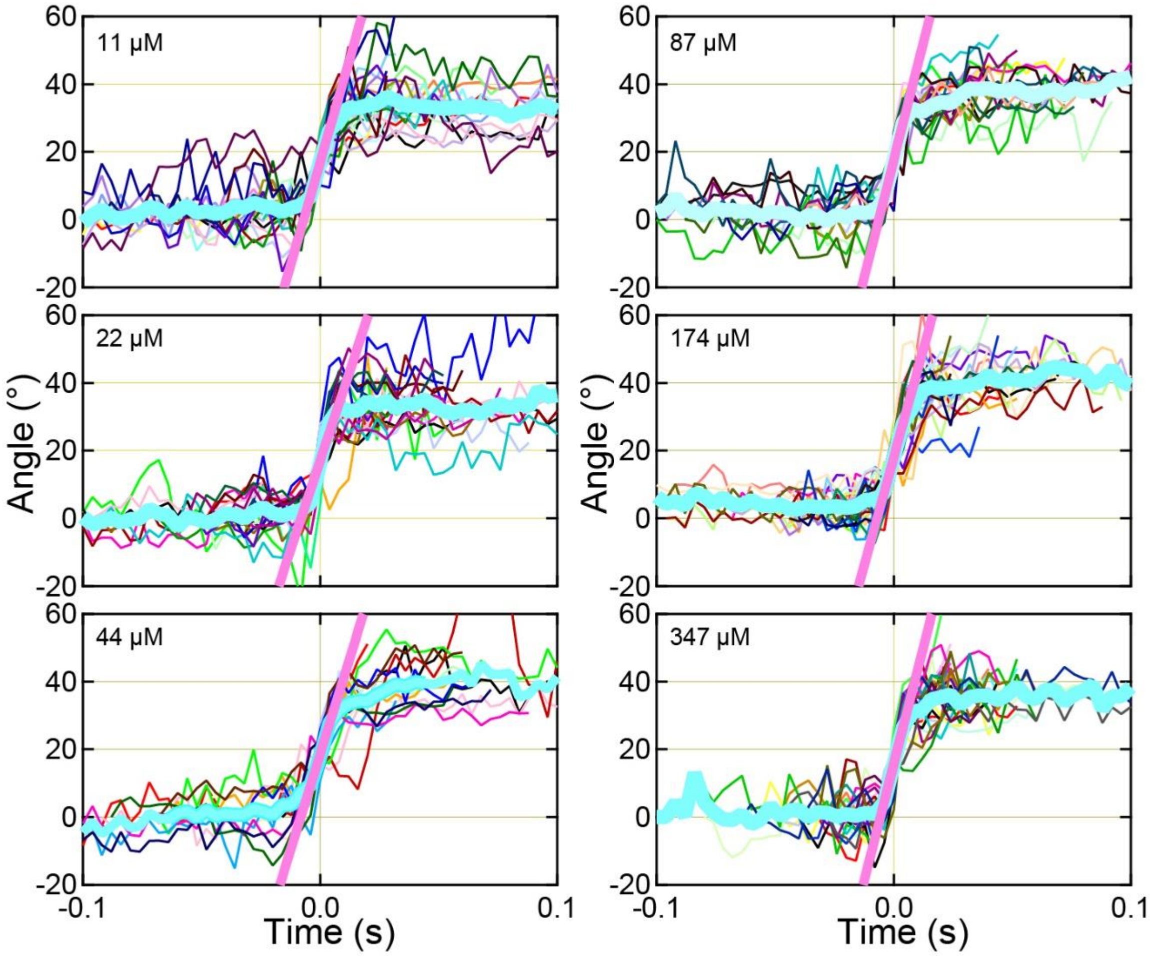

Supplementary Figure 5 Stepping torque

Thin colored curves represent the raw data with the size of 30–40° steps; thick blue curves are their average (n = 26, 21, 18, 25, 26, 30 consecutive steps at 11 μM, 22 μM, 44 μM, 87 μM, 174 μM, and 347 μM [ATP]). Time zero for each step record was assigned closest to 15°. Pink thick lines represent the linear fit to each averaged trace between 4° and 30 °, providing the torque. The slopes at 11 μM, 22 μM, 44 μM, 87 μM, 174 μM, and 347 μM [ATP] were 42.64 rad s^{-1}, 36.92 rad s^{-1}, 39.63 rad s^{-1}, 48.55 rad s^{-1}, 44.70 rad s^{-1}, and 48.2 rad s^{-1}, respectively, which corresponded to the angular velocity. With an angular velocity and the viscous friction of the single ghost (2.4 pN nm s), the stepping torque was estimated to be 85–120 pN nm.

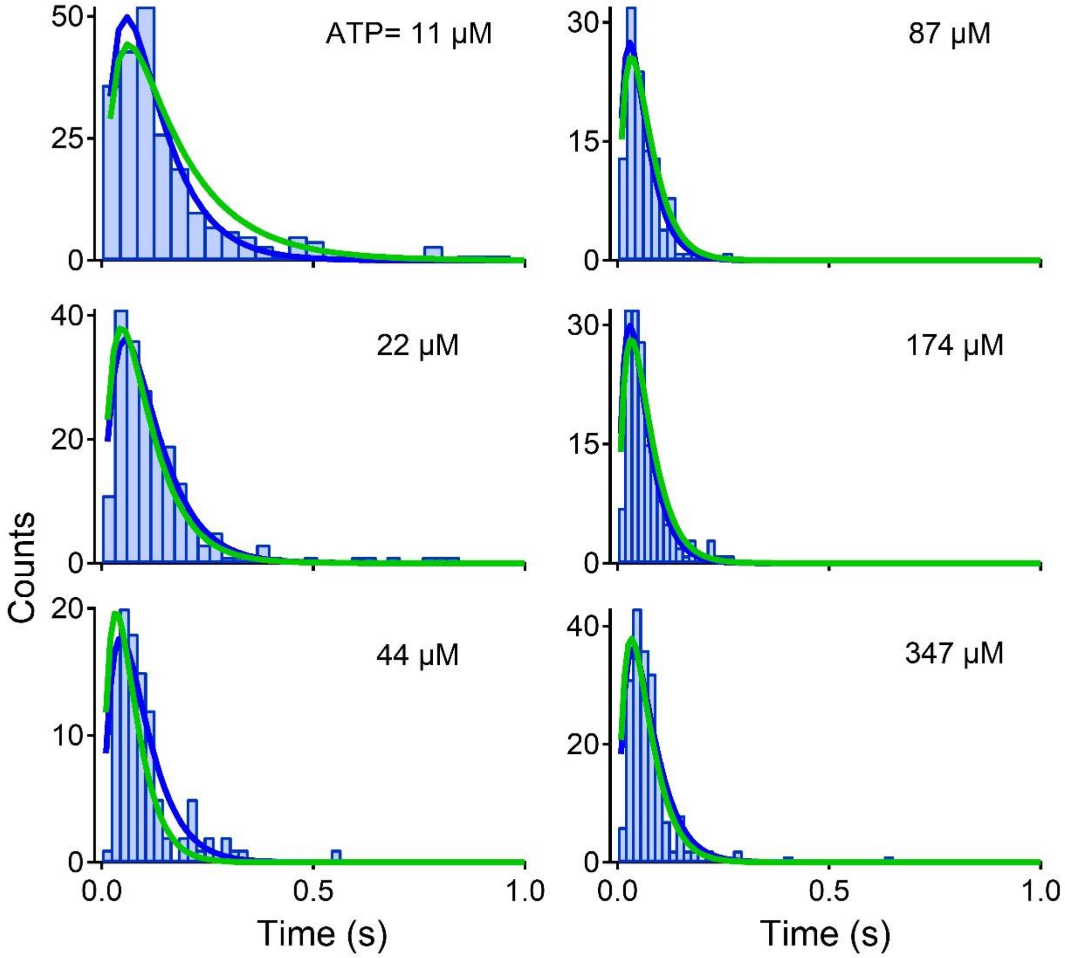

Supplementary Figure 6 Dwell time analysis

Histograms of dwell times at various [ATP]s. Total counts are 233, 197, 105, 111, 156 and 193 steps in the range of 11–347 μM [ATP]s. Blue lines are double exponential fits: constant·(exp(-k_1 × t)-exp(-k_2 × t)), where k_1 and k_2 represent green and pink filled circle in Fig. 4c. Note that $k_1$and k_2 were changed above 44 μM ATP. Green line are global fitting: constant·(exp(-(k_{on} × [ATP] × t)-(exp(-k_1 × t) for 11-44 μM ATP and constant·(exp(-k_1 × t)-exp(-k_2 × t)) for 87–347μM ATP , where k_{on} was the rate constant for ATP binding (6.7×10^5 M^{-1} s^{-1}) and two ATP-independent reaction with k_1 and k_2 were 29.6 $s^{-1}$and 31.9 s^{-1} .

Captions for Supplementary Videos

Movie S1

Rotational motion of ghost at a saturated [ATP] observed by a phase-contrast microscopy with a temporal resolution of 33 ms. Scale bar, 10 μm.

Movie S2

A propeller rotation at 8 μM [ATP]. The ghost had the rotation axis at the middle and rotated like a propeller Scale bar, 1 μm

Movie S3

Inhibition of a rotational motion by sialyllactose. Sialyllactose was added into the chamber around 10 sec. Scale bar, 10 μm.

Movie S4

Inhibition of a rotational motion by 0.5 mg ml^{-1} antibody MabR19 which influenced on the crank protein, Gli521. Scale bar, 20 μm.
